# Single-Quantum Sodium MRI at 3T for the Separation of Mono- and Bi-T2 Sodium Signals

**DOI:** 10.21203/rs.3.rs-5456028/v1

**Published:** 2025-04-07

**Authors:** Yongxian Qian, Ying-Chia Lin, Xingye Chen, Yulin Ge, Yvonne W. Lui, Fernando E. Boada

**Affiliations:** 1 Bernard and Irene Schwartz Center for Biomedical Imaging, Department of Radiology, New York University Grossman School of Medicine, New York, NY 10016.; 2 Neuroscience Institute, New York University Grossman School of Medicine, New York, NY 10016.; 3 Vilcek Institute of Graduate Biomedical Sciences, NYU Grossman School of Medicine, New York, NY 10016.; 4 Department of Radiology, NYU Langone Health, New York, NY 10016.

## Abstract

Sodium magnetic resonance imaging (MRI) is highly sensitive to cellular ionic balance due to tenfold difference in sodium concentration across membranes, actively maintained by the sodium-potassium (Na^+^-K^+^) pump. Disruptions in this pump or membrane integrity, as seen in neurological disorders like epilepsy, multiple sclerosis, bipolar disease, and mild traumatic brain injury, lead to increased intracellular sodium. However, this cellular-level alteration is often masked by the dominant extracellular sodium signal, making it challenging to distinguish sodium populations with mono- vs. bi-exponential transverse (T_2_) decays – especially given the low signal-to-noise ratio (SNR) even at an advanced clinical field of 3 Tesla. Here, we propose a novel technique that leverages intrinsic difference in T_2_ decays by acquiring single-quantum images at multiple echo times (TEs) and applying voxel-wise matrix inversion for accurate signal separation. Using numerical models, agar phantoms, and human subjects, we achieved high separation accuracy in phantoms (95.8% for mono-T_2_ and 72.5–80.4% for bi-T_2_) and demonstrated clinical feasibility in humans. This approach may enable early detection of neurological disorders and early assessment of treatment responses at the cellular level using sodium MRI at 3T.

## Introduction

In human brains, sodium ions (Na^+^), when exposed to an electric field gradient of negatively charged macromolecules and proteins, experience nuclear quadrupolar interaction that results in biexponential decay in transverse (T_2_) relaxation of nuclear spins when ions are not in fast motion – a situation in which correlation time between sodium ions and electric field gradient is much shorter than the inverse of the Larmor frequency, $\tau_{c}\ll 1∕\omega_{0}$ [1, 2]. On the other hand, sodium ions in relatively fast motion cancel out the effect of quadrupolar interactions, resulting in mono-exponential T_2_ decay [1-4]. Historically, the short-T_2_ component of the biexponential decay was mis-considered arising from “bound” sodium [5, 6] as it was not detectable by then-NMR (nuclear magnetic resonance) [1-4]. The terminology of “bound sodium” however remains in use in today’s literature of sodium MRI, but it comes into question more recently [7]. For clarity, this article refers to the “bi-T_2_” sodium population as those ions showing biexponential T_2_ decay and “mono-T_2_” sodium population as those ions showing mono-exponential T_2_ decay. It is important to note that mono- and bi-T_2_ sodium ions can be present in both intra- and extracellular spaces [8-10], dependent on relative correlation time with electric field gradient [1, 2].

Sodium (^23^Na) MRI (magnetic resonance imaging) currently acquires signals from both populations of sodium ions (mono- and bi-T_2_) and quantifies total sodium concentration (TSC) at each voxel. TSC is a unique MRI measure that taps ionic status in tissue and has the potential to non-invasively detect disruptions in ionic homeostasis present in a variety of conditions including stroke, tumor, demyelination, epilepsy, bipolar disorder, and mild traumatic brain injury [8-12]. However, TSC can be dominated by the mono-T_2_ sodium population in the cerebrospinal fluid (CSF) of a high sodium concentration (~145 mM) and overshadows alterations happening in the intracellular space of a much lower concentration (~15 mM), particularly in the gray matter due to its proximity to the brain CSF spaces. Separation of mono- and bi-T_2_ sodium signals would effectively remove the impact of CSF and more clearly depict intracellular alterations, especially at the early stage of a disease happening at the cellular level or in the early (cellular) response to a treatment.

The difference in T_2_ relaxation was extensively explored in sodium MRI to separate the two populations of sodium ions in the brain. Triple quantum filtering (TQF) in which magnetic resonance (MR) signals are generated solely from triple-quantum (TQ) transitions, was considered a standard for human studies [13]. TQF techniques, however, require multiple radiofrequency (RF) pulses for excitation and multi-step phase cycling to extract TQ signals and eliminate single-quantum (SQ) signals [13-17], leading to long scan times (20–40 min) and high specific absorption rate (SAR) – the two major concerns about safety and practicality. More problematic is that the TQF signal has very low signal-to-noise ratio (SNR), about tenfold lower than the SQ [15-17]. These difficulties hamper translatable use of TQF in human subjects. Recent efforts relaxed the SAR concern by using two or even one RF pulse, but the low SNR issue remained [24, 52, 53].

Alternative approaches have also been proposed. Inversion recovery (IR), adopted from proton (^1^H) MRI, exploits the difference in longitudinal (T_1_) relaxation between mono- and bi-T_2_ sodium ions, and attempts to suppresses signals from mono-T_2_ sodium of longer T_1_ time [18-20]. Such IR approaches require an additional RF pulse for the suppression, further compounding the SAR issue [20, 21], although adiabatic pulses are usually used. It also suffers from incomplete suppression of mono-T_2_ sodium signals due to spatial inhomogeneities of the ${B_{1}}^{+}$ field and from partial loss of the desired bi-T_2_ sodium signal due to incomplete recovery at the chosen inversion time. Incomplete suppression further complicates accurate quantification of bi-T_2_ sodium due to unknown residual mono-T_2_ sodium signals which is tenfold higher than bi-T2 sodium signal [9,11,18]. To overcome these drawbacks, another alternative approach, called the short-T_2_ imaging, was proposed in which SQ images were acquired at multiple echo times (TEs) and subtracted from each other to produce an image of the short-T_2_ component of bi-T_2_ sodium [22-25]. In such a way, SAR was reduced to, and SNR was increased to, the level of SQ images, favorable to human studies in clinic. Unfortunately, the subtraction could not eliminate completely mono-T_2_ sodium signal (~20% in residual signal), degrading accuracy of bi-T_2_ sodium quantification [25].

In this study, the concept of the short-T_2_ imaging is generalized to multi-TE single-quantum (MSQ) imaging with more accurate matrix inversion replacing the subtraction to substantially improve accuracy of the separation between mono- and bi-T_2_ sodium signals. To develop the MSQ technique, we optimized TEs for data acquisition, investigated impact of T_2_ values on accuracy of the separation, and acquired free induction decay (FID) signals to generate a T_2_* spectrum for aiding the matrix equation. To test the MSQ technique, we implemented numerical simulations, phantom experiments, and human studies. The results are supportive of the proposed MSQ technique. We also itemized limitations of the MSQ technique and potential pitfalls in the interpretation of the separated sodium signals.

## Results

### Theoretical development

#### Model of sodium MRI signals

We use a two-population model, equation (1), to describe the single-quantum sodium image signal, m(t), evolving with time t at an imaging voxel ΔV. Time t=0 is at the center of excitation RF pulse.


(1) $$m(t)=m_{mo}\;Y_{mo}(t)+m_{bi}\;Y_{bi}(t),\;t\geq 0\;\;\text{with}\;m_{mo}\geq 0,\;m_{bi}\geq 0,\;\text{and}\;m_{mo}+m_{bi}=m(0)$$

(1a) $$Y_{mo}(t)\equiv \text{exp}\;(-t∕T_{2,mo})$$

(1b) $$Y_{bi}(t)\equiv a_{bs}\;\text{exp}\;(-t∕T_{2,bs})+a_{bl}\;\text{exp}\;(-t∕T_{2,bl})\;.$$

The $m_{mo}$ and $m_{bi}$ are signal intensity proportional to volume fraction $v_{q}$ and sodium concentration $C_{q}$, i.e., $m_{q}\propto \Delta Vv_{q}C_{q}$, q=mo and bi, for mono-T_2_ and bi-T_2_ sodium populations in a voxel ΔV, respectively. m(0) is total sodium signal intensity. $Y_{mo}(t)$ is the relaxation decay of the mono-T_2_ sodium with time constant $T_{2,mo}$ while $Y_{bi}(t)$ corresponds to the bi-T_2_ sodium with $a_{bs}=0.6$ for the short-T_2_ component $T_{2,bs}$ and $a_{bl}=0.4$ for the long-T_2_ component $T_{2,bl}$. The ratio 0.6:0.4 in intensity of the bi-T_2_ sodium is from theoretical and experimental results for sodium nuclear spins in single molecular environment [1-4] and is applicable to some types of biological tissues with multiple molecular environments, such as rabbit brains and kidneys [5] and implanted brain tumors in rats [26]. The values of $T_{2}$ are in an order of $T_{2,bs}\ll T_{2,bl}\leq T_{2,mo}$. However, equation (1) doesn’t include mono-T_2_ sodium with a short T_2_ value.

#### Separation of mono- and bi-T_2_ sodium signals

We then found a solution to equation (1) and separated the mono- and bi-T_2_ sodium signal intensities, $m_{mo}$ and $m_{bi}$. Given SQ sodium images at multiple echo times, $\text{TEs}=\{TE_{1},TE_{2},\ldots ,TE_{N}\}$, in which TE is defined as time interval between the center of excitation RF pulse and center of the k-space, equation (1) becomes a matrix equation below.


(2) M=YX

(2a) $$M\equiv {\left(m_{1},m_{2},\ldots ,m_{N}\right)}^{T},\;m_{i}=m(TE_{i}),i=1,2,\ldots ,N$$

(2b) $$X\equiv {\left(m_{mo},m_{bi}\right)}^{T}$$

(2c) $$Y\equiv \left(\begin{array}{cc} Y_{mo}(TE_{1}) & Y_{bi}(TE_{1})\\ Y_{mo}(TE_{2}) & Y_{bi}(TE_{2})\\ \vdots & \vdots \\ Y_{mo}(TE_{N}) & Y_{bi}(TE_{N}) \end{array}\right)$$

Where the superscript T is operator for matrix transpose. A solution to equation (2) is given in equation (3) via an established algorithm called non-negative least-squares (NNLS) [27] in which the non-negative condition on X is incorporated into the solution.


(3) X=NNLS(YX−M)

**Fig. 1.**
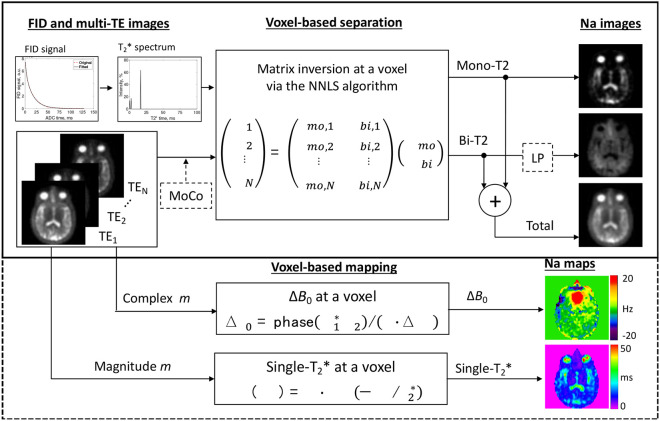
Flowchart of the proposed MSQ sodium MRI. **Input:** multi-TE SQ images m(TE) and an FID signal producing T_2_* spectrum. Motion correction (MoCo) between SQ images is optional. **Separation:** voxel-wise matrix inversion. **Output:** three sodium images, i.e., mono-T_2_ as $m_{\text{mo}}$, bi-T_2_ as $m_{\text{bi}}$, and total as $m_{\text{mo}}+m_{\text{bi}}$. The low-pass (LP) filtering (optional) is a 3D smoothing of size 3×3×3 or others, to further reduce random noise. **Additional outputs:** the maps of $B_{0}$ inhomogeneity and single-T_2_*.

We summarized this solution, called the MSQ approach, into a flowchart in Fig. 1. The inputs are multi-TE sodium images and an FID signal. The outputs are mono-T_2_, bi-T_2_, and total sodium images, as well as maps of field inhomogeneity $\Delta B_{0}$ and single-term exponential fitted effective T_2_ (called T_2_*) – single-T_2_* for short. Hereinafter, T_2_* replaces T_2_ as spin echo is not favorable to sodium MRI. Motion correction (MoCo) across multi-TE images is optional. Also optional is the low-pass (LP) filtering, which is a 3D averaging over a volume of 3×3×3 voxels for instance, to reduce random noise on the bi-T_2_ sodium images. The $\Delta B_{0}$ and single-T_2_* maps represent spatial distributions of the $B_{0}$ field inhomogeneity and the short- and long-T_2_* components, providing indications for uncertain short-T_2_* decays possibly caused by $B_{0}$ inhomogeneity. Such maps are complimentary but critical to quantification and interpretation of the separated mono- and bi-T_2_ sodium signals.

### Technical development

#### Optimization of the number of TEs

In principle, the more TEs the better differentiation between T_2_* relaxations of the mono- and bi-T_2_ sodium populations. In practice, the number of TEs is restricted by total scan time (TA), SNR, signal decay, and risk of motion across TEs. Therefore, a trade-off must be made for the number of TEs. To determine an optimal number of TEs, it is necessary to understand noise propagation in equation (2). We applied singular value decomposition (SVD) analysis [28] to the matrix Y in equation (2) and got equation (3)

(3) $$Y=U\;\Sigma \;V^{T}$$

(3a) $$\Sigma =\text{diag}(\sigma_{1},\sigma_{2})$$

(3b) $$X=V\;\Sigma^{-1}\;U^{T}\;M\;.$$

Singular values ($\sigma_{1}\geq \sigma_{2}\geq 0$) determine noise transfer (amplification or suppression) in equation (3b) from the measured TE-images M to the separated mono- and bi-T_2_ sodium images X. However, equation (3b) allows negative values in X when random noise contaminates M. This violates the “non-negative” condition on X. Therefore, the SVD analysis is applicable only to X elements with SNR ≥ 2 where the elements, with Gaussian noise, have 95.4% of chance in the territory of non-negative value [29].

The simulations were implemented via equation (3) for three cases: an idea case serving as reference, practical case 1 having a large number of TEs, and practical case 2 having a small number of TEs. The idea case has 80 TEs, i.e., TE=(0,1,2,…,79), to cover entire range of the T_2_* decays. The two practical cases, based on our extensive experience [18,23,28,33] on human studies, have total scan time limited to 22min for 8 TE-images, 2.66min for each TE-image; and the distribution of TEs was chosen such that it was most sensitive to T_2_* decays [23]. Thus, the case 1 had 8 TEs, i.e., TE = (0.5, 1, 2, 3, 4, 5, 7, 10) ms, while the case 2 only has two TEs, i.e., TE = (0.5, 5.0) ms but four averages at each TE. The 4-averages lead to a reduction in random noise standard deviation (SD), or an increase in SNR, by a factor of 2 in the case 2 relative to case 1. The optimal set of TEs is identified as the one that has a $\sigma_{2}$ value producing minimum amplification of random noise in the separated mono- and bi-T_2_ sodium images.

**Fig. 2.**
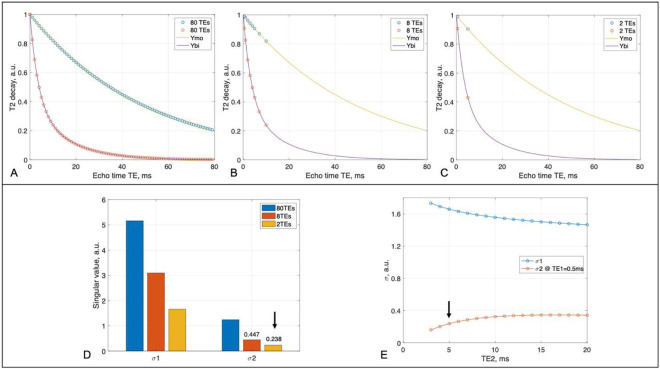
Optimization of TE sampling scheme. **(A)** A reference sampling scheme of 80 TEs in a range of 0–79 ms at an interval of 1.0 ms, distributing on a mono-exponential T_2_* decay $Y_{\text{mo}}$ (TE) of mono-T_2_ sodium at a typical value of ${{T_{2}}^{*}}_{\text{mo}}=50\;\text{ms}$, and on a bi-exponential T_2_* decay $Y_{\text{bi}}$ (TE) of bi-T_2_ sodium at a typical set ${{T_{2}}^{*}}_{\text{bs}},{{T_{2}}^{*}}_{\text{bl}}\}=\{3.5,15.0\}\;\text{ms}$. **(B)** The 8-TE case at TE={0.5, 1, 2, 3, 4, 5, 7, 10} ms, distributing on the decay curves $Y_{\text{mo}}$ and $Y_{\text{bi}}$. **(C)** The 2-TE case at TE={0.5, 5} ms. **(D)** SVD singular values ($\sigma_{1}$ and $\sigma_{2}$) of the three sampling schemes. $\sigma_{2}$ is less than 1.0 at both 8- and 2-TE schemes but the 2-TEs (arrow) leads to less noise amplification when using four averages. **(E)** Singular values of the 2-TE scheme changing with TE_2_ at 5ms (arrow) produced a value near maximum for $\sigma_{2}$ while preserving higher signal than the larger TE_2_. Collectively, the 2-TEs scheme is an optimal one for the human brain.

Fig. 2 presents the results at a typical set of T_2_* values, $\{T_{2,mo}^{*},T_{2,bs}^{*},T_{2,bl}^{*}\}=\{50.0,3.5,15.0\}\;\text{ms}$. Singular value $\sigma_{2}$ is less than 1.0 for the 8-TE and 2-TE cases, leading to a noise amplification. Therefore, a better choice for less noise amplification is the 2-TE case, in which TE_2_ at 5ms produced a value near maximum of $\sigma_{2}$ while preserving higher signal than larger TE_2_. Thus, the 2-TE scheme was selected for our studies.

#### Impact of T_2_* values on the separation

Intuitively, the solution $\left\{m_{mo},m_{bi}\right\}$ to equation (1) is not sensitive to T_2_* values due to exponential decays in equations (1a,b). This observation can be verified theoretically and numerically Theoretically, small changes in T_2_* values, $\left\{\delta T_{2,q}^{*},q=mo,bs,bl\right\}$, lead to small changes $\left\{\delta m_{p},p=mo,bi\right\}$ in $m_{mo}$ and $m_{bi}$ under the same m(t), that is.

(4) $$0=\delta (m_{mo}Y_{mo})+\delta (m_{bi}Y_{bi})$$

(4a) $$-m_{\delta }=Y_{mo}\delta m_{mo}+Y_{bi}\delta m_{bi}$$

(4b) $$m_{\delta }\equiv m_{mo}\delta Y_{mo}+m_{bi}\delta Y_{bi}$$

(4c) $$\delta Y_{q}=\left(\frac{tY_{q}}{T_{2,q}^{*}}\right)\left(\frac{\delta T_{2,q}^{*}}{T_{2,q}^{*}}\right)\leq e^{-1}\left(\frac{\delta T_{2,q}^{*}}{T_{2,q}^{*}}\right),\;\;q=mo,\;bs,\;bl$$

(4d) $$\delta Y_{bi}=a_{bs}\;\delta Y_{bs}+a_{bl}\;\delta Y_{bl}\leq e^{-1}\left[a_{bs}\left(\frac{\delta T_{2,bs}^{*}}{T_{2,bs}^{*}}\right)+a_{bl}\left(\frac{\delta T_{2,bl}^{*}}{T_{2,bl}^{*}}\right)\right]$$

where δ is difference operator. Numerically, errors in $m_{mo}$ and $m_{bi}$ can be calculated, given a series of $\left\{T_{2,q}^{*},\delta T_{2,q}^{*};q=mo,bs,bl\right\}$ at a specific pair $\left(m_{mo},m_{bi}\right)$. This creates a plot showing how $\left\{m_{mo},m_{bi}\right\}$ changes with $\left\{\delta T_{2,q}^{*};q=mo,bs,bl\right\}$.

**Fig. 3.**
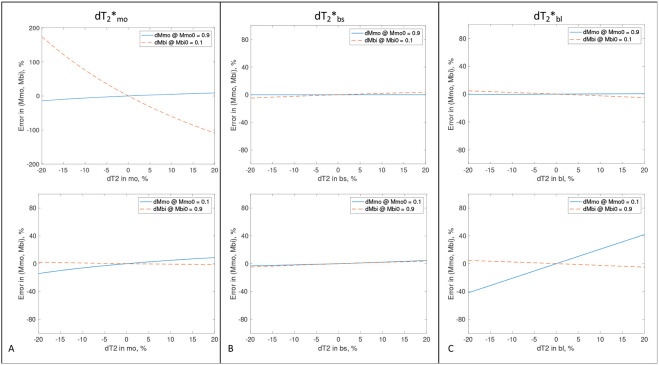
Impact of T_2_* values on the separation of mono- and bi-T_2_ sodium signals $\left(m_{\text{mo}},m_{\text{bi}}\right)$ at $\{{{T_{2}}^{*}}_{\text{mo}},{{T_{2}}^{*}}_{\text{bs}},{{T_{2}}^{*}}_{\text{bl}}\}=(50.0,3.5,15.0)\;\text{ms}$ for two extreme cases: mono-T_2_ sodium dominating (top row), $m_{\text{mo}}=0.9$, and bi-T_2_ sodium dominating (bottom row), $m_{\text{bi}}=0.9$. **(A)** Error in $T_{2}{*}_{\text{mo}}$. **(B)** Error in $T_{2}{*}_{\text{bs}}$. **(C)** Error in $T_{2}{*}_{\text{bl}}$.

Fig. 3 shows two extreme cases: mono-T_2_ sodium dominating at $m_{mo}=0.9$ and bi-T_2_ sodium dominating at $m_{bi}=0.9$. In Fig. 3a, error in $T_{2,mo}^{*}$ causes an error in $m_{mo}$ or $m_{bi}$ much smaller for the dominant one, e.g., $\delta m_{bi}<2.2\%$ when $\delta T_{2,mo}^{*}<20\%$, and $\delta m_{mo}<2.9\%$ when $\delta T_{2,mo}^{*}<5.0\%$. In Fig. 3b, error in $T_{2,bs}^{*}$ has a small impact on both $m_{mo}$ and $m_{bi}$, e.g., when dominating, $\delta m_{bi}<4.8\%$ and $\delta m_{mo}<0.04\%$ when $\delta T_{2,bs}^{*}<20\%$. In Fig. 3c, error in $T_{2,bl}^{*}$ leads to an error in $m_{mo}$ or $m_{bi}$ much smaller for the dominant one, e.g., $\delta m_{bi}<5.2\%$ and $\delta m_{mo}<0.6\%$ when $\delta T_{2,bl}^{*}<20\%$. The best case is Fig. 3b where $T_{2,bs}^{*}$ has small impact (<4.9%) on both mono- and bi-T_2_ sodium signals. The worst case is Fig. 3a (top) where $T_{2,mo}^{*}$ had a large impact on bi-T_2_ sodium signal, $\delta m_{bi}=35.6\%$ when $\delta T_{2,mo}^{*}=\text{-}5\%$. In other words, when the mono-T_2_ sodium is very dominating, $T_{2,mo}^{*}$ value should be as accurate as possible (achievable via single-T_2_* map) to attain the best separation for the bi-T_2_ sodium.

### Testing on numerical and physical models

#### Numerical models

**Fig. 4.**
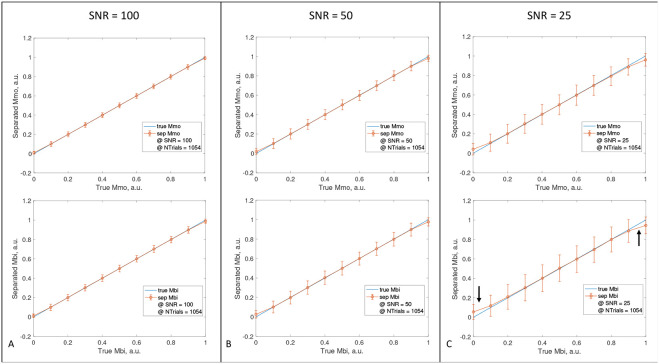
Simulated separation of the mono- and bi-T_2_ sodium signals. **(A)** Extra-high SNR = 100. **(B)** High SNR = 50. **(C)** Regular SNR = 25. The SD (error bar) of the separated $m_{\text{mo}}$ (top) and $m_{\text{bi}}$ (bottom) consistently decreases with SNR increasing from 25 to 100, with an underestimate (arrow) for $m_{\text{mo}}$ or $m_{\text{bi}}$ near maximum value 1.0 but an overestimate (arrow) near minimum value 0.0. The amount decreases with SNR increasing.

We first tested the separation of mono- and bi-T_2_ sodium signals on numerical models via equation (2) at a typical set of T_2_* values, $(T_{2,mo}^{*},T_{2,bs}^{*},T_{2,bl}^{*})=(50.0,3.5,15.0)\;\text{ms}$, and an optimal two-TE scheme, TE = (0.5, 5.0) ms. Sodium image signals were calculated via equation (1), with an additive Gaussian noise, $N(0,\sigma^{2})$, at each of noise trials (independent from each other), m(t)+n(t). The mono- and bi-T_2_ signal intensities $\left\{m_{mo},m_{bi}\right\}$ vary in a normalized range of 0.1–0.9 at a step size = 0.1. The separation was implemented using the function lsqnonneg() in MATLAB, and repeated $N_{\text{noise}}$ times at each of the specific intensity. Mean and standard deviation (SD) were reported as the separated sodium signal. $N_{\text{noise}}=1054$ was chosen to detect a 10% of SD, or 0.1 effect size d=Δμ∕SD, in difference between the mean and true value at 90% power and 5% significant level under the two-sided Student’s *t*-test [29]. Fig. 4 demonstrates the simulated separation at three SNRs of interest: 100 (extra-high), 50 (high), and 25 (regular). The SD (error bar) consistently decreased with SNR increasing. There were underestimates (3.9–5.6%) in $m_{mo}$ and $m_{bi}$ near maximum value 1.0 but overestimates (4.2–5.5%) near minimum value 0.0, with the amount decreasing with SNR increasing.

#### Physical models

We then tested the separation on four physical models (phantoms) with known sodium concentrations. These phantoms were custom-built and described in our previous publications [25, 30]. They are 50-mL centrifuge tubes filled with a mixture of distilled water, 10% w/w agar powder, and sodium chloride (NaCl) at three concentrations of 90, 120, and 150 mM and at 150mM without agar, to mimic bi- and mono-T_2_ sodium signals in human brains. Sodium MRI was performed on a clinical MRI scanner at 3T (MAGNETOM Trio Tim, Siemens Medical Solutions, Erlangen, Germany) with a dual-tuned (^1^H-^23^Na) volume head coil (Advanced Imaging Research, Cleveland, OH). The data acquisition was implemented using an SNR-efficient, three-dimensional (3D) pulse sequence called twisted projection imaging (TPI) [31].

**Fig. 5.**
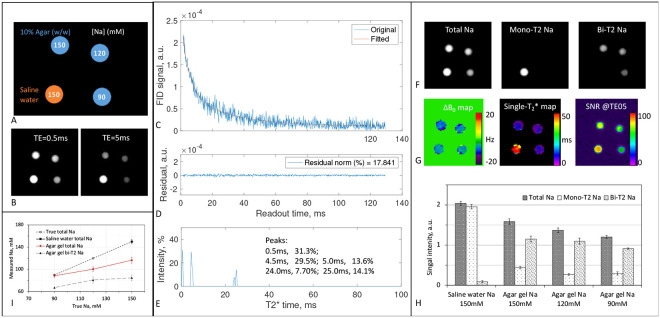
Phantom study. **(A)** Phantoms of four tubes with sodium concentration: 150mM for the saline water simulating mono-T_2_ sodium ions and 90, 120, 150 mM for the agar gels simulating bi-T_2_ sodium ions. **(B)** Sodium images of the phantoms at TE_1_/TE_2_=0.5/5ms, shown in the same window/level. **(C)** FID signals (original and fitted) from the four tubes (no averaging), with the correction for distortion at the first five data points. **(D)** Residual error of the fitting in C. **(E)** T_2_* spectrum calculated from the FID in C and used to produce the fitted FID. **(F)** Sodium images (total, mono-T_2_, and bi-T_2_) separated from the two images in B at $({{T_{2}}^{*}}_{\text{mo}},{{T_{2}}^{*}}_{\text{bs}},{{T_{2}}^{*}}_{\text{bl}})=(50,5,25)\;\text{ms}$ according to E and G. **(G)** Maps of $\Delta B_{0}$ and single-T_2_* calculated from the images in B, and a map of SNR at TE=0.5ms. **(H)** Separated sodium signals in the regions of tubes in F. **(I)** Quantified sodium concentration from F.

Fig. 5 summarizes outcomes of the phantom studies [30]. The separation in Fig. 5h recovered 95.8% of mono-T_2_ sodium signal in the saline water tube, while leaving 4.2% to bi-T_2_ sodium signal, much better than 20% left by the subtraction approach [25]. The separation recovered 72.5, 80.4, and 75.9 % of bi-T_2_ sodium signal in the agar tubes at sodium concentrations of 150, 120, and 90mM, respectively. The quantification of sodium concentration in Fig. 5i, when calibrated at the saline water, showed a systematic bias in total and bi-T_2_ sodium concentrations, leading to an underestimate of sodium concentrations.

### Testing on humans

To demonstrate feasibility of the proposed MSQ approach at clinical field strength of 3 Tesla, we conducted the human study on 15 subjects including nine healthy adults (age 39.6±21.4 years in a range of 21–74 years; 3 males and 6 females) and six patients with different neurological conditions (1 bipolar disorder, 3 epilepsy, 1 multiple sclerosis, and 1 mild traumatic brain injury; age 30.5±15.1 years in a range of 18–59 years; 3 males and 3 females), after the exclusion of one healthy subject and one patient due to motion between the two TE-images. The MSQ sodium MRI was performed on a clinical 3T MRI scanner (Magnetom Prisma, Siemens Healthineers, Erlangen, Germany) with a custom-built 8-channel dual-tuned (^1^H-^23^Na) head array coil [32].

#### T_2_* values in whole brain of the study subjects

The MSQ approach needs an input of $\left\{T_{2,mo}^{*},T_{2,bs}^{*},T_{2,bl}^{*}\right\}$ at *each* voxel ΔV in the field of view (FOV). To measure these values is time consuming (~2 hours) and impractical in clinical routine. Alternatively, we proposed to use a global set of these values for *all* voxels. This global set can be measured quickly (<1 min) on whole brain of each study subject by acquiring FID signal s(t) and fitting to it with multi-term exponential T_2_* decays to attain a T_2_* spectrum through equation (5).


(5) $$|s(t)|=\sum_{j}A_{j}\;\text{exp}\;(-t∕T_{2,j}^{*})$$

**Fig. 6.**
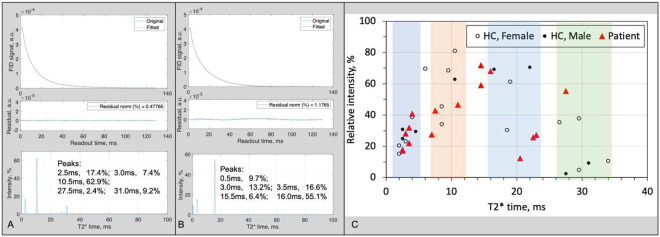
Human study: individual T_2_* components in whole brain across all the 15 study subjects. **(A)** A typical T_2_* spectrum and associated FID signal and fitting error from whole brain of a healthy young subject (21 years old, male). **(B)** Another example from a patient with epilepsy (31 years old, male). **(C)** Scatter plot of individual T_2_* components.

To confirm the validity of such alternative approach, we acquired FID signals on all the 15 subjects. Fig. 6 demonstrates two such FID signals and T_2_* spectra from a healthy young subject (21 years old, male, Fig. 6a) and a patient with epilepsy (31 years old, male, Fig 6b). The spectra are sparse with just 2–4 peaks, indicating that the global set of $\left\{T_{2,mo}^{*},T_{2,bs}^{*},T_{2,bl}^{*}\right\}$ is a reasonable estimate for the separation. We also observed that these T_2_* values however are different from subject to subject as shown in the two-dimensional (2D) scatter plot (Fig. 6c). The short-T_2_* component is clearly crowded in a range of 1–5 ms while the long-T_2_* is widely scattered in three bands centered at 10, 20, and 30 ms, respectively. Interestingly, the long-T_2_* component is shifted to lower values in the patient group, relative to the healthy group, potentially due to micro magnetic environment altered in the patients. There seems no difference between males and females in the healthy group.

#### Healthy subjects

**Fig. 7.**
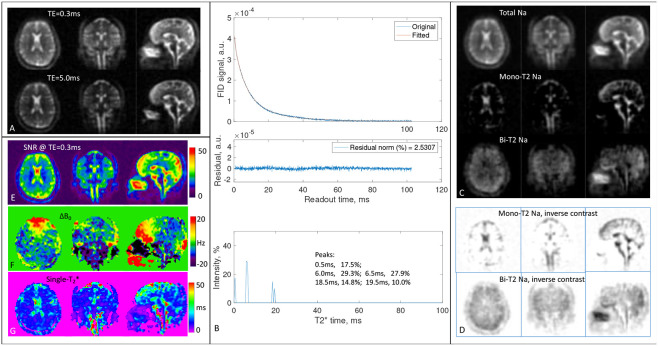
Human study #1 (26-year-old female, healthy). **(A)** 3D sodium images of the brain in three orthogonal slices at TE_1_/TE_2_=0.3/5ms. **(B)** FID signal of whole brain and the associated fitting error and T_2_* spectrum. **(C)** Separated sodium images from the 2-TE images in A using $({{T_{2}}^{*}}_{\text{mo}},{{T_{2}}^{*}}_{\text{bs}},{{T_{2}}^{*}}_{\text{bl}})=(50.0,6.0,19.0)\;\text{ms}$ according to peaks in B. **(D)** Inverse-contrast display to highlight areas of low intensity. All the images in A and C were displayed using the same window/level. **(E-G)** Maps of SNR, $\Delta B_{0}$, and single-T_2_*, calculated from the 2-TE images in A.

**Fig. 8.**
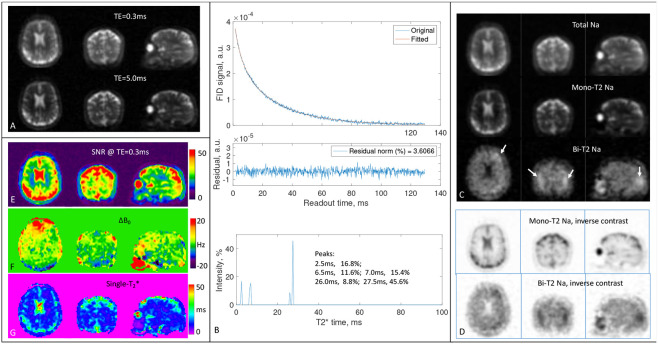
Human study #2 (59-year-old male, patient with bipolar disorder). **(A)** 3D sodium images of the brain at TE_1_/TE_2_=0.3/5ms. **(B)** FID signal of whole brain and the associated fitting error and T_2_* spectrum. **(C)** Separated sodium images from the 2-TE images in A using $({{T_{2}}^{*}}_{\text{mo}},{{T_{2}}^{*}}_{\text{bs}},{{T_{2}}^{*}}_{\text{bl}})=(50.0,2.5,7.0)\;\text{ms}$ according to the peaks in B. **(D)** Inverse-contrast display to highlight areas of low intensity. All the images in A and C were displayed using the same window/level, except bi-T2 sodium where W/L was halved. **(E-G)** Maps of SNR, $\Delta B_{0}$, and single-T_2_*, calculated from the 2-TE images in A. Note that the bi-T_2_ sodium images in C highlight possible regions (arrows) of elevated bi-T_2_ sodium concentration in the parenchyma.

We presented here two representative cases of the 15 human subjects: a healthy young subject (26 years old, female) in Fig. 7 and a mid-aged patient with self-reported bipolar disorder (59 years old, male) in Fig. 8. The other cases are available to readers under the data availability conditions.

Fig. 7 indicates that signals from CSF in the brain were effectively separated into mono-T_2_ sodium image (Fig. 7c or d), while signals from brain tissues such as gray and white matters were mostly contained in the bi-T_2_ sodium image (Fig. 7c or d). Notably and not surprisingly, signal intensity across brain tissues looks more uniform in the bi-T_2_ sodium images than in the mono-T_2_ sodium images (Fig. 7c), total sodium images (Fig. 7c), or TE_1_-images (Fig. 7a), which is in consistency with the knowledge that the bi-T_2_ sodium ions are mainly located in intracellular space of the brain tissues with a normal concentration of about 15 mM [5, 26]. SNR in Fig. 7e is ≥ 25 in most regions of the brain, ensuring a robust separation as suggested in the simulations (Fig. 4). The field inhomogeneity $\Delta B_{0}$ in Fig. 7f varied between ±20 Hz across the brain, with the largest off-resonance in the prefrontal and occipital lobes, leading to visible blurring of the tissues in the bi-T_2_ sodium images (Fig. 7c or d, sagittal). The single-T_2_* map in Fig. 7g provides a spatial distribution of short and long T_2_* components across the brain, complementary to T_2_* spectrum in Fig. 7b. It also indicates that majority of the long T_2_* components are in the prefrontal lobe in this case (Fig. 7g, sagittal).

Fig. 8 shows potential benefits from the bi-T_2_ sodium images of patients with neurological disorders such as bipolar disorder which is known to cause abnormally high intracellular sodium concentration in the brain, but locations are hard to define [33, 34]. The bi-T_2_ sodium images (Fig. 8c or d) clearly highlighted brain regions of an elevated bi-T_2_ sodium signal against surrounding tissues, with a ratio of 1.78 vs. 1.40 (or 27.1% increase) before the separation (Fig. 8c). These regions have no visible contrast in the total or TE_1_-images (Fig. 8a or c). SNR in these regions is > 40 (Fig. 8e), supporting a robust separation. The field inhomogeneity $\Delta B_{0}$ in these regions is low (<5 Hz, Fig. 8f), excluding field-induced artifacts. Single-T_2_* map in Fig. 8g shows abnormally low T_2_* values in the regions, confirming an increase in short-T_2_* components.

#### Validation via the estimates of upper limit for extra- and intracellular volume fractions

The human studies, different from the phantom studies above where the ground truth of sodium concentrations is known, lack the ground truth for validation. Although each step in the MSQ separation is scientifically reasonable and its outcome should be believed correct, we alternatively and indirectly validate it by applying the separated sodium images to estimation of the upper limit of extra- and intracellular volume fractions for which accurate measurements are available in literatures [8, 26, 35, 36]. To that goal, we assigned all mono-T_2_ sodium to extracellular space and all bi-T_2_ sodium to intracellular space, although they may co-exist in both extra- and intracellular spaces. The estimates (mean and SD) were made in the regions of interest (ROIs) of gray matter (GM) and white matter (WM) across three continuous slices via equations (6-8) using sodium concentrations $C_{ex}=145\text{mM}$ for extracellular space and $C_{in}=15\text{mM}$ for intracellular space.


(6) $$v_{ex}=1∕(1+a)$$

(7) $$v_{in}=a∕(1+a)$$

(8) $$a=m_{bi}C_{ex}∕m_{mo}C_{in}$$

**Fig. 9.**
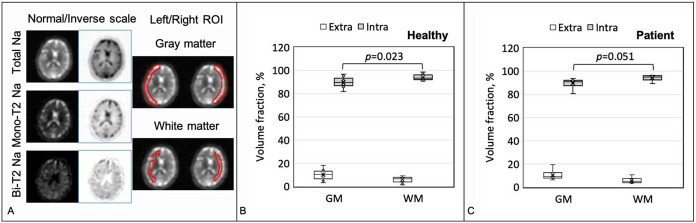
Estimates of upper limit of the volume fraction for extra- and intracellular spaces. **(A)** Typical sodium images (total, mono-T_2_, and bi-T_2_) and ROIs for the gray matter (GM) and white matter (WM) in an axial slice from a healthy subject. **(B)** Volume fractions for the healthy group N=9), and **(C)** for the patient group (N=6).

Fig. 9 presents the upper-limit estimates in the healthy (N=9) and patient (N=6) groups. The healthy group shows significant (P=0.023) difference in volume fraction between the gray and white matters: 89.6±4.5 vs. 94.0±2.6 % for intracellular space, in line with the literatures of 75 vs. 92 % [8, 26]; and 10.4±4.5 vs. 6.0±2.6 % for extracellular space, comparable to literatures of 14.1±1.8 [35] vs. 18±5 % [36]. There’s no significant difference (P=0.953) between the healthy and patient groups.

## Discussion

We presented here a new technique, MSQ, that simply separates the mono- and bi-T_2_ sodium signals with high accuracy offered by voxel-wise matrix inversion (Fig. 1). The MSQ approach leverages intrinsic difference in T_2_ relaxation between the two sodium populations (Figs. 7, 8). The 2-TE sampling scheme stands out for smaller noise transfer during the separation (Fig. 2). The T_2_* spectrum of whole brain has sparse peaks and confirms a global set of T_2_* values $\left\{T_{2,mo}^{*},T_{2,bs}^{*},T_{2,bl}^{*}\right\}$ applicable to humans (Fig. 6). The measurement of T_2_* spectrum facilitates fine tuning of the global set of T_2_* values towards individual subjects with diverse T_2_* relaxations in the brain (Figs. 7, 8). However, a global set of T_2_* values may not be plausible in such situations where T_2_* in an individual brain has substantial spatial variation [37-44], multi-regional sets, or a linear combination of them, may be applied to the separation.

This paper outlines a novel method for sodium signal separation and, of note, does not detail subsequent quantification of sodium concentrations of the two sodium populations after separation. Such quantification involves further procedures including calibration to sodium concentration from signal using different strategies such as external phantoms or internal regions like ventricles and vitreous humors and corrections for inhomogeneous coil sensitivity including the RF fields of ${B_{1}}^{+}$ and ${B_{1}}^{\text{-}}$. These procedures merit further study.

Limitations of the MSQ approach are important to understand when applying them. First, the method assumes a two-population model exhibiting mono- and bi-T_2_ decay behaviors. Theoretically, this could be a problem if, for example, there is no bi-T_2_ signal at all but rather two mono-T_2_ sodium decays of different T_2_* values within the same voxel; these components would be mathematically combined and then falsely created as what appears to a bi-T_2_ sodium population. This error stems from the fact that the described separation is based on a *mathematical* model, rather than the *physical* model as the TQF approaches are based on. To minimize this kind of false positive errors, the NNLS algorithm, rather than the analytical one, is required to solve the matrix equation (2) under the non-negative constrains although it takes longer time in computation.

The second limitation may occur at voxels filled solely with mono-T_2_ sodium of very small T_2_* values, such as regions in the paranasal sinuses (Figs. 7c and 8c). Such areas may mislead the MSQ to produce pseudo bi-T_2_ sodium. Any such mis-separated regions can be readily identified by cross-referencing the maps of $\Delta B_{0}$ and single-T_2_* (Figs. 7 and 8).

The third limitation is potential underestimation of bi-T_2_ sodium signal caused by the image at TE_1_, as illustrated in the phantom studies (Fig. 5). The separation in equation (2) assumes TE_1_-image intensity precisely at TE_1_ (i.e., a very short readout time). Actual TE_1_-image intensity is an average over readout time during which short-T_2_* components decay significantly when readout is relatively long (e.g., $T_{s}=36.32\text{ms}$, which is about ten times longer than the short- T_2_* of 3ms seen in this study). Therefore, the extent of underestimation depends on readout time or pulse sequence. To mitigate this problem, two strategies may be applicable. One is to replace TE_1_ in equation (2) with an effective (larger) value accounting for short-T_2_* decay during readout. The other is to shift $T_{2,bs}^{*}$ to a larger value, as did in a previous work on phantoms [30]. Alternatively, correction for such underestimation could be integrated into calibration process when quantifying sodium concentration (Fig. 5).

## Materials and methods

### Experimental Design

The objective of this study is to demonstrate the feasibly of the proposed MSQ approach to separate mono- and bi-T_2_ sodium signals in brain tissues of human subjects at a clinical field strength of 3 Tesla. We designed three types of experiments: numerical simulations, phantom experiments, and human subject studies. To implement the MSQ approach, we used a clinical MRI scanner at 3 Tesla for data acquisition and a custom-developed software package for data processing.

### Calculation of the separation sensitivity to T_2_* values

We used the most common set of T_2_* values from our human studies, $\{T_{2,mo}^{*},T_{2,bs}^{*},T_{2,bl}^{*}\}=\{50.0,3.5,15.0\}\text{ms}$, as true values, and calculate via equations (1-3) the separation error $\left\{\delta m_{mo},\delta m_{bi}\right\}$ relative to the ground truth of mono- and bi-T_2_ sodium signals $\left\{m_{mo},m_{bi}\right\}$ when adding errors $\delta T_{2,q}^{*}$, q=mo, bs, bl, up to ±20% to the true T_2_* values. The relationship between $\left\{\delta T_{2,q}^{*};q=mo,bs,bl\right\}$ and $\left\{\delta m_{mo},\delta m_{bi}\right\}$ was plotted out in Extended Data Fig. E2. To focus on the relation of $‘‘\delta T_{2}^{*}-\delta m’’$, TEs were sampled in an ideal case with ${\text{TE}}_{0}=0$,ΔTE=1.0ms, and 80 TEs covering entire T_2_* decays.

### Numerical simulation

These simulations were performed in MATLAB on the MacBook Pro laptop or Windows desktop, otherwise specified. Random noise of Gaussian distribution was generated using MATLAB function randn(n), while the NNLS algorithm was implemented using [x,resnorm,residual]=lsqnonneg(C,d).

### Phantom experiments

We custom-built four phantoms, which were detailed in our previous work [25]. The phantoms are 50-mL centrifuge tubes filled with a mixture of distilled water, 10% w/w agar powder, and sodium chloride (NaCl) at three concentrations (90, 120, and 150 mM) and at 150mM without agar, mimicking bi- and mono-T_2_ sodium signals in brain tissues. MRI data acquisition was implemented using an SNR-efficient, three-dimensional (3D) pulse sequence called twisted projection imaging (TPI) [31], with parameters: rectangular RF pulse duration = 0.8ms, flip angle=80° (limited by SAR and TR), field of view (FOV)=220mm, matrix size=64, nominal resolution=3.44mm (3D isotropic), TPI readout time=36.32ms, total TPI projections=1596, TPI p-factor=0.4, TR=100ms, TE_1_/TE_2_= 0.5/5ms, averages=4, and TA=10.64min per TE-image. The sodium images were offline reconstructed on a desktop computer (OptiPlex 7050, 8GB memory, Windows 10, DELL, Round Rock, TX) using the gridding algorithm [47, 48] and a custom-developed programs in C++ (MS Visual Studio 2012, Microsoft, Redmond, WA). The mono- and bi-T_2_ sodium signals was separated using a custom-developed program as described above.

### Clinical MRI scanner for data acquisition

The sodium MRI images and FID signals were acquired on two 3T clinical MRI scanners for the phantom experiments and human studies, respectively. One was MAGNETOM Trio Tim (Siemens Medical Solutions, Erlangen, Germany) with a dual-tuned (^1^H-^23^Na) volume head coil (Advanced Imaging Research, Cleveland, OH) and used for the phantom experiments. The other was MAGNETON Prisma (Siemens Healthineers, Erlangen, Germany) with a custom-built 8-channel dual-tuned (^1^H-^23^Na) head array coil [32] and used for human studies.

### Software for data processing and image display

We developed a software (code) called *SepMoBi* in MATLAB (R2021a, MathWorks, Natick, MA) on a laptop computer (MacBook Pro, 16GB memory, Apple M1 chip, Apple Inc., Cupertino, CA), and used it for data processing, including the calculations of T_2_* spectrum, mono- and bi-T_2_ sodium images, and maps of SNR, $\Delta B_{0}$ and single-T_2_*. In addition, we used software *MRView* (MRI Research, Mayo Clinic and Foundation, December 2020) for image display and parameter calculation in ROIs (Fig. 9).

### Pre-processing of FID signals

When acquired with an array coil, FID signals may have unique initial phases $\left\{\varphi_{0,l},l=1,2,\ldots ,N_{c}\right\}$ at individual elements, and need to be aligned to produce a resultant FID signal. Alignment (via phase correction) can be towards a reference phase such as zero phase, one of the initial phases, or mean phase across elements. In addition, signal intensity at individual elements needs to be scaled using “FFT factor” stored in the header of a raw FID data file, for instance.

FID signals at the first few samples are distorted by hardware filtering during analog-to-digital conversion (ADC). The number of affected samples are in a range of 3–10 points, dependent on sampling bandwidth, with the first sample having the largest distortion. This distortion alters measurement of T_2_* components, especially the short T_2_* components which are critically important to the bi-T_2_ sodium. Correction for the distortion can be performed using an established exponential extrapolation (see Supporting Materials).

### Global T_2_* spectrum

We measured FID signals on clinical 3T MRI scanners using a product pulse sequence, either *AdjXFre* embedded in manual shimming or independent *fid_23Na,* with acquisition parameters: TE=0.35–1.0ms, TR=100–300ms, and averages =1–128, and TA=0.2–39s. After the pre-processing described above, resultant FID signals are curve-fitted to equation (5) using the NNLS algorithm [27] when T_2_* values are pre-distributed in a range of interest $\left[{{T_{2}}^{*}}_{\text{min}},{{T_{2}}^{*}}_{\max}\right]$ at uniform or non-uniform intervals $\left\{\Delta T_{2,j}^{*},j=1,2,\ldots \right\}$. Amplitudes $\left\{A_{j}\right\}$, called T_2_* spectrum, determine relative incidence of T_2_* components in an imaging volume which counts all T_2_* components from both mono- and bi-T_2_ sodium populations. We used a uniform interval of $\Delta T_{2}^{*}=0.5\text{ms}$ in a range of interest 0.5–100 ms for a high resolution of T_2_* values. Assignment of the peaks in the T_2_* spectrum to $\left\{T_{2,mo}^{*},T_{2,bs}^{*},T_{2,bl}^{*}\right\}$ was based on their relative positions of $T_{2,mo}^{*}\geq T_{2,bl}^{*}\gg T_{2,bs}^{*}$ and intensity ratio about 6:4 for the bi-T_2_ sodium. Robustness of the T_2_* measurement including data acquisition and spectrum computation is addressed in the Supporting Information. In case of missing $T_{2,mo}^{*}$ peak in the T_2_* spectrum representing cerebrospinal fluid (CSF), use the single-T_2_* map to determine the value of $T_{2,mo}^{*}$ by calculating the mean value in a region filled with pure CSF in the ventricles or with pure fluid in the vitreous humors.

### Human studies

The human studies were approved by local Institutional Review Board (IRB) at New York University Grossman School of Medicine, New York, NY, USA, and performed in accordance with relevant guidelines and regulations. Informed consent was obtained from each of the study subjects. For data acquisition, the same TPI pulse sequence was used as in the phantom studies. Sodium images were reconstructed off-line using the gridding algorithm [47, 48], channel by channel, and combined into a resultant image via the sum-of-squares (SOS) algorithm [49]. To decouple random noise across channels, an orthogonal linear transform (detailed in Ref. 46) was performed in which physical channel data were transformed into virtual channels with random noise independent from channel to channel. This decoupling and denoising process also normalized signal amplitudes across channels by dividing noise standard deviation. Separation of the mono- and bi-T_2_ sodium signals was then implemented in the same way as in the phantom studies.

### Mapping of $\Delta B_{0}$ and single-T_2_*

To map $\Delta B_{0}$ or $\Delta f_{0}(=\gamma \Delta B_{0}∕2\pi )$, Hermitian product method [50] was performed via equations (9-11) at individual imaging voxels to calculate phase differences $\left\{\Delta \varphi_{i},i=1,2,\ldots ,N-1\right\}$ between $\text{TEs}\;\{TE_{i},i=1,2,\;\ldots ,N\}$. Image amplitude at individual channels were corrected with the scale factors $\left\{w_{l},l=1,2,\ldots ,N_{c}\right\}$. Phase unwrapping was not performed due to small intervals in the TEs and, in general, small inhomogeneity in $B_{0}$ field in sodium MRI. Computation for $\Delta B_{0}$ map is fast (0.078s) on a Mac laptop computer for images of matrix size 64×64×64 at two TEs.


(9) $$\Delta f_{0}=\frac{1}{2\pi (N-1)}\sum_{i=1}^{N-1}\Delta \varphi_{i}∕\Delta TE_{i}$$

(10) $$\Delta \varphi_{i}=phase\{\sum_{l=1}^{Nc}w_{l}^{2}\cdot m_{l}^{*}\;(TE_{i})\cdot m_{l}(TE_{i+1})\}$$

(11) $$\Delta TE_{i}=TE_{i+1}-TE_{i}$$

To map single-T_2_*, a MATLAB curve-fitting function fit(x,y,‘exp1’) was used to calculate single-T_2_* values at each voxel via equation (12). A restriction (${{T_{2}}^{*}}_{\max}<100\text{ms}$) was enforced to exclude unreasonable values caused by random noise. The computation time is acceptable (10min17s).


(12) $$|m(TE_{i})|=A_{0}\text{exp}\;(-TE_{i}∕T_{2}^{*})\;,\;0\leq T_{2}^{*}\leq T_{2,max}^{*}$$

### Calculation of signal-to-noise ratio (SNR)

In a region of interest (ROI), SNR was calculated via equation (13) in a simplified way for both volume and array coils by taking the ratio of mean intensity S in a ROI to noise standard deviation (SD) in noise-only background regions. A factor of 0.655 was applied to noise SD to account for Rician distribution in magnitude images [51]. For SNR mapping, pixel signal is used in the calculation.


(13) SNR=0.655S∕SD

### Statistical Analysis

A regular statistical significance (P=0.05) was applied to the comparisons, via Student𠅩s *t*-test, between the two sets of data in this work. Minimum sample size for the *t*-test is 16, with 80% power, 5% significance level, two-sided test, and 1.0 effect size [29].
